# Quizartinib, a selective FLT3 inhibitor, maintains antileukemic activity in preclinical models of RAS-mediated midostaurin-resistant acute myeloid leukemia cells

**DOI:** 10.18632/oncotarget.27489

**Published:** 2020-03-17

**Authors:** Tomoya Aikawa, Noriko Togashi, Koichi Iwanaga, Hiroyuki Okada, Yumi Nishiya, Shinichi Inoue, Mark J. Levis, Takeshi Isoyama

**Affiliations:** ^1^Daiichi Sankyo Co., Ltd., Tokyo, Japan; ^2^The Sidney Kimmel Comprehensive Cancer Center at Johns Hopkins University, Baltimore, MD, United States of America

**Keywords:** relapsed/refractory AML, FLT3 inhibitors, quizartinib, AC886, midostaurin resistance

## Abstract

*FLT3* internal tandem duplication (ITD) mutations are associated with poor prognosis in patients with acute myeloid leukemia (AML). In this preclinical study, we characterized the binding affinity and selectivity of quizartinib, a small-molecule inhibitor of FLT3, and AC886, the active metabolite of quizartinib, compared with those of other FLT3 inhibitors. Selectivity profiling against >400 kinases showed that quizartinib and AC886 were highly selective against FLT3. Quizartinib and AC886 inhibited FLT3 signaling pathways in *FLT3*-ITD–mutated AML cells, leading to potent growth inhibition with IC_50_ values of <1 nM. When quizartinib was administered to mice bearing *FLT3*-ITD mutated tumors, AC886 was rapidly detected and tumor regression was observed at doses of ≥1 mg/kg without severe body weight loss. In addition, quizartinib inhibited the viability of midostaurin-resistant MOLM-14 cells and exerted potent antitumor activity in mouse xenograft models without severe body weight loss, while midostaurin and gilteritinib did not show significant antitumor effects. This is the first detailed characterization of quizartinib and AC886 in comparison with other FLT3 inhibitors under the same experimental conditions. Preclinical antileukemic activity in midostaurin-resistant *FLT3*-ITD–mutated AML cells suggests the potential value of quizartinib following midostaurin failure in patients with *FLT3*-ITD mutated AML.

## INTRODUCTION

FMS like tyrosine kinase 3 (FLT3) is a receptor tyrosine kinase expressed by acute myeloid leukemia (AML) cells in 70% to 90% of patients [1]. *FLT3* mutations have been found in approximately 30% of AML cases, with internal tandem duplication (ITD), commonly found in the juxtamembrane domain of FLT3, occurring in approximately 25% of cases and mutations in the tyrosine kinase domain (TKD) present in approximately 5% [2–5]. *FLT3*-ITD is the most common form of *FLT3* mutation and is associated with a higher rate of relapse and poorer clinical outcomes [6, 7], while *FLT3*-TKD mutations have not been definitively linked to poor prognosis [8]. The *FLT3*-ITD mutations constitutively activate FLT3 signaling, acting through the PI3 kinase/AKT and MEK/ERK pathways, as well as STAT5, both from the cell membrane and as an immature form originating from the endoplasmic reticulum [9–11].

FLT3 tyrosine kinase inhibitors are classified into 2 categories. Type I, including midostaurin, gilteritinib, and crenolanib, which bind the active conformation of FLT3, and type II, including quizartinib and sorafenib, which bind the inactive conformation [12, 13]. FLT3 inhibitors vary considerably in kinase selectivity [14]. Generally, type II inhibitors are more selective than type I inhibitors, as the inactive conformation preferred by type II inhibitors is thought to be more kinase specific than the active conformation [15]. It is speculated that highly selective FLT3 inhibitors can be administered at lower doses, improving tolerability and minimizing off-target effects. The type II FLT3 inhibitor quizartinib demonstrated high kinase selectivity [16], but its active metabolite AC886 has not been characterized.

AML relapse is associated with the accumulation of additional genetic mutations and clonal evolution, which is shaped by the primary treatment regimen received [17]. Secondary kinase mutations can emerge in *FLT3*-ITD AML, resulting in resistance to FLT3 inhibitors, and point mutations that confer resistance to a certain FLT3 inhibitor tend to have cross-resistance to other drugs in the same class [18].

Agents that treat AML by targeting FLT3 are beginning to be approved, yet limited options remain. Midostaurin (RYDAPT^®^) is the only approved FLT3 inhibitor for newly diagnosed *FLT3* mutation–positive AML and is administered in combination with standard cytarabine and daunorubicin induction and cytarabine consolidation [19, 20]. Gilteritinib (XOSPATA^®^) was recently approved for the treatment of relapsed or refractory *FLT3* mutation–positive AML [21–23]. Quizartinib prolonged survival in patients with relapsed/refractory *FLT3*-ITD–positive AML compared with salvage chemotherapy [24], and quizartinib (VANFLYTA^®^) was recently approved in Japan. Limited data exist on the efficacy of available FLT3 inhibitors in patients with AML that was relapsed or refractory to a first-line midostaurin-based therapy, and strategies to overcome resistance mutations, such as a combination of inhibitors or use of more potent FLT3 inhibitors, are being evaluated [25].

The objectives of this preclinical study were to characterize the kinase binding affinity and selectivity of quizartinib and its active metabolite AC886 compared with those of other FLT3 inhibitors, to evaluate the antitumor effect of quizartinib on midostaurin-resistant AML cells, and to assess the impact of midostaurin resistance on FLT3 inhibitors.

## RESULTS

### Quizartinib and its active metabolite AC886 bound to FLT3 with high affinity and selectivity

**Figure 1:**
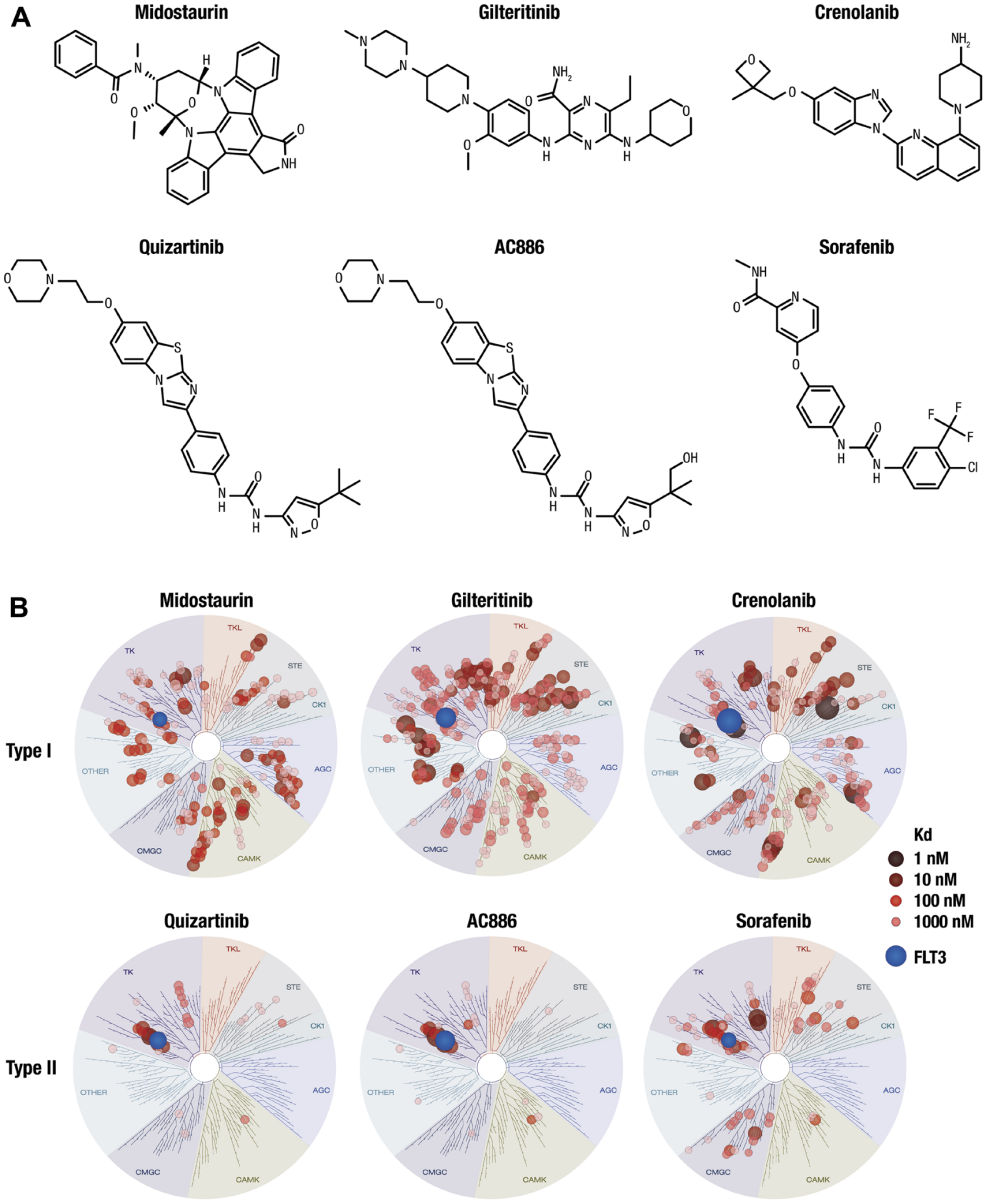
Chemical structure and kinase selectivity profiling of quizartinib, AC886, and other FLT3 inhibitors. (**A**) Chemical structures of midostaurin, gilteritinib, crenolanib, quizartinib, AC886, and sorafenib. (**B**) Binding affinity of compounds was measured against 404 nonmutant kinases. Kd values were calculated from duplicate 11-point dose-response curves. Each red circle represents a kinase bound to each compound. Larger circles indicate higher binding affinity of each compound to kinases. Kinases with Kd values <3000 nM are shown. Blue circles represent FLT3.

The binding affinities of the type I FLT3 inhibitors midostaurin, gilteritinib, and crenolanib and type II FLT3 inhibitors sorafenib, quizartinib, and its metabolite AC886 (Figure 1A) were evaluated under the same experimental conditions against a panel of 404 nonmutant kinases. Both quizartinib and its active metabolite AC886 bound to FLT3 with high affinity, with Kd values of 3.3 and 1.1 nM, respectively (Figure 1B; Supplementary Table 1; Supplementary Appendix). The Kd values of other FLT3 inhibitors for FLT3 binding were 7.9 nM (midostaurin), 1.0 nM (gilteritinib), 0.28 nM (crenolanib), and 5.9 nM (sorafenib) (Figure 1; Supplementary Table 1; Supplementary Appendix).

Quizartinib was highly selective; among the 404 nonmutant kinases evaluated, quizartinib bound to FLT3 with the greatest affinity and bound only 2 kinases with Kd <10 nM (FLT3 and KIT). In addition to FLT3, quizartinib and AC886 each bound 7 other kinases with Kd <100 nM, while the other inhibitors evaluated bound between 19 and 83 other kinases with that affinity (Table 1; Supplementary Appendix). Kinases that were bound by FLT3 inhibitors with Kd <1 nM were KIT for AC886; ALK for gilteritinib; and MEK5, PDGFRα, and ULK2 for crenolanib. To assess off-target inhibition, the inhibitory activities of quizartinib and AC886 were assessed against a panel of 87 off-target molecules such as G-protein–coupled receptors, transporters, ion channels, nuclear receptors, and enzymes. Quizartinib and AC886 showed >50% inhibition against only 2 and 3 molecules, respectively, at a high concentration of 10 µM, suggesting that these compounds had minimal off-target inhibitory activity (Supplementary Table 2).

**Table 1:** Kinase selectivity profiling of FLT3 inhibitors

Kd value	Number of kinases					
	Quizartinib	AC886	Midostaurin	Gilteritinib	Crenolanib	Sorafenib
<1 nM	0	1	0	1	4	0
≥1 and <10 nM	2	2	5	16	9	4
≥10 and <100 nM	6	5	49	67	40	16
≥100 and <1000 nM	15	10	83	107	78	30

### Quizartinib and AC886 had similarly potent and durable inhibition of FLT3 signaling in *FLT3*-ITD AML cells

**Figure 2:**
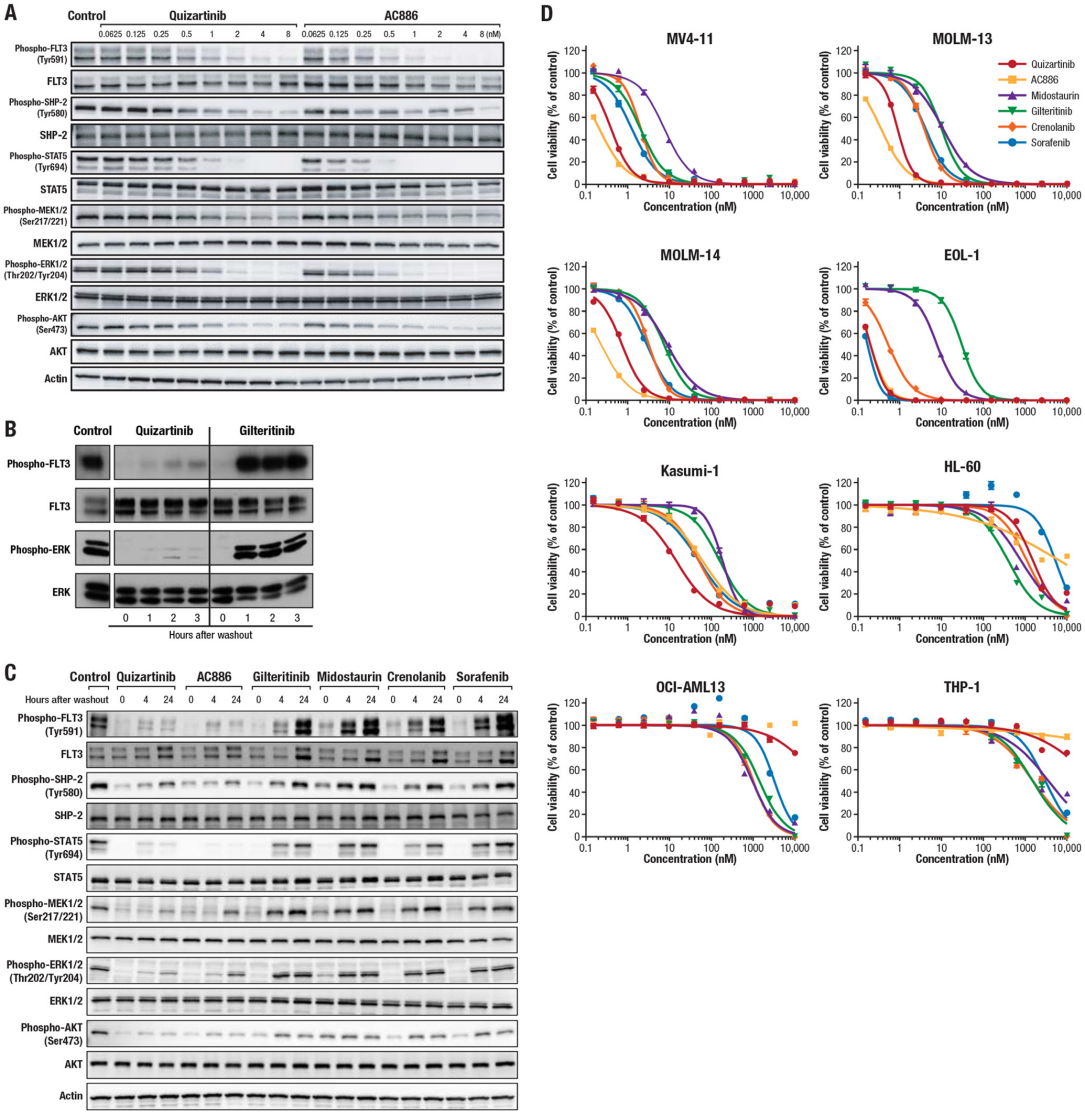
Inhibitory activity of quizartinib and other FLT3 inhibitors on the FLT3 signaling pathway and the viability of AML cells. (**A**) MV4-11 cells were treated with quizartinib or AC886 at the indicated concentrations for 2 hours. Western blot analysis was performed on cell lysates using antibodies against the indicated proteins. (**B–C**) MOLM-14 cells were incubated with FLT3 inhibitors at 20 nM or with DMSO control for (B) 1 or (C) 2 hours. Cells were pelleted, washed, and resuspended in medium and incubated for (B) up to 3 hours or (C) up to 24 hours. (B) Western blot analysis was performed on FLT3 immunoprecipitants (FLT3) and whole-cell lysates (ERK). (C) Western blot analysis was performed on cell lysates using antibodies against the indicated proteins. (**D**) AML cells were incubated with each FLT3 inhibitor for 3 days. Cell viability was assessed using the CellTiter-Glo 2.0 Assay (Promega Corporation, Madison, Wisconsin, USA) and plotted at each concentration. Each data point and bar represent the mean and standard deviation of each concentration group, respectively (*n* = 3).

Quizartinib and AC886 had concentration-dependent inhibitory activity on the FLT3 signaling pathway in the AML cell line MV4-11, which harbors the *FLT3*-ITD mutation (Figure 2A). Quizartinib potently inhibited FLT3 phosphorylation (50% inhibitory concentration [IC_50_], 0.50 nM), and AC886 showed greater inhibition of FLT3 phosphorylation at lower concentrations, with an IC_50_ value of 0.18 nM. Quizartinib and AC886 similarly inhibited phosphorylation of downstream molecules SHP-2, STAT5, MEK1/2, ERK1/2, and AKT (Figure 2A).

It has previously been reported that quizartinib has a slow dissociation rate from FLT3, leading to prolonged inhibition of FLT3 signaling for up to 24 hours after compound withdrawal in MV4-11 cells [26]. To assess the durability of signaling inhibition by FLT3 inhibitors in *FLT3*-ITD MOLM-14 cells, cells were treated with quizartinib or gilteritinib at 20 nM for 1 hour, and then the levels of phosphorylated FLT3 and ERK were measured up to 3 hours after removal of the inhibitors from the cells. Quizartinib maintained inhibition of FLT3 phosphorylation and ERK phosphorylation through 3 hours after removal, while gilteritinib had no significant inhibitory activity starting 1 hour after removal (Figure 2B). We also assessed the long-term durability of FLT3 inhibitors, and the results indicated that quizartinib and AC886 maintained inhibition of FLT3 signaling for up to 24 hours after compound withdrawal, while midostaurin, gilteritinib, crenolanib, and sorafenib were no longer able to inhibit FLT3 signaling at 24 hours (Figure 2C).

### *In vitro* inhibitory activity of quizartinib and AC886 on the viability of *FLT3*-ITD AML cells

We examined the growth inhibitory activity of the FLT3 inhibitors on both *FLT3*-ITD and wild-type *FLT3* AML cells. Quizartinib reduced the viability of the *FLT3*-ITD AML MV4-11, MOLM-13, and MOLM-14 cells, with IC_50_ values of 0.40, 0.89, and 0.73 nM, respectively (Supplementary Table 3). AC886 had similar impact on *FLT3*-ITD AML cell viability, with IC_50_ values for MV4-11, MOLM-13, and MOLM-14 cells of 0.21, 0.36, and 0.23 nM, respectively (Supplementary Table 3). Both quizartinib and AC886 potently inhibited the growth of wild-type *FLT3* AML EOL-1 (with FIP1L-PDGFRα) cells and moderately inhibited growth of Kasumi-1 (with KIT N822K) cells, but they had minimal impact on the growth of other wild-type *FLT3* AML cells. Midostaurin, gilteritinib, crenolanib, and sorafenib all had less inhibitory activity on the 3 *FLT3*-ITD cell lines than both quizartinib and AC886, suggesting that quizartinib and AC886 exert potent antileukemic activity selectively on *FLT3*-ITD AML (Figure 2D; Supplementary Table 3; Supplementary Figure 1).

### *In vivo* efficacy of quizartinib and AC886 vs other FLT3 inhibitors in a mouse xenograft model

**Figure 3:**
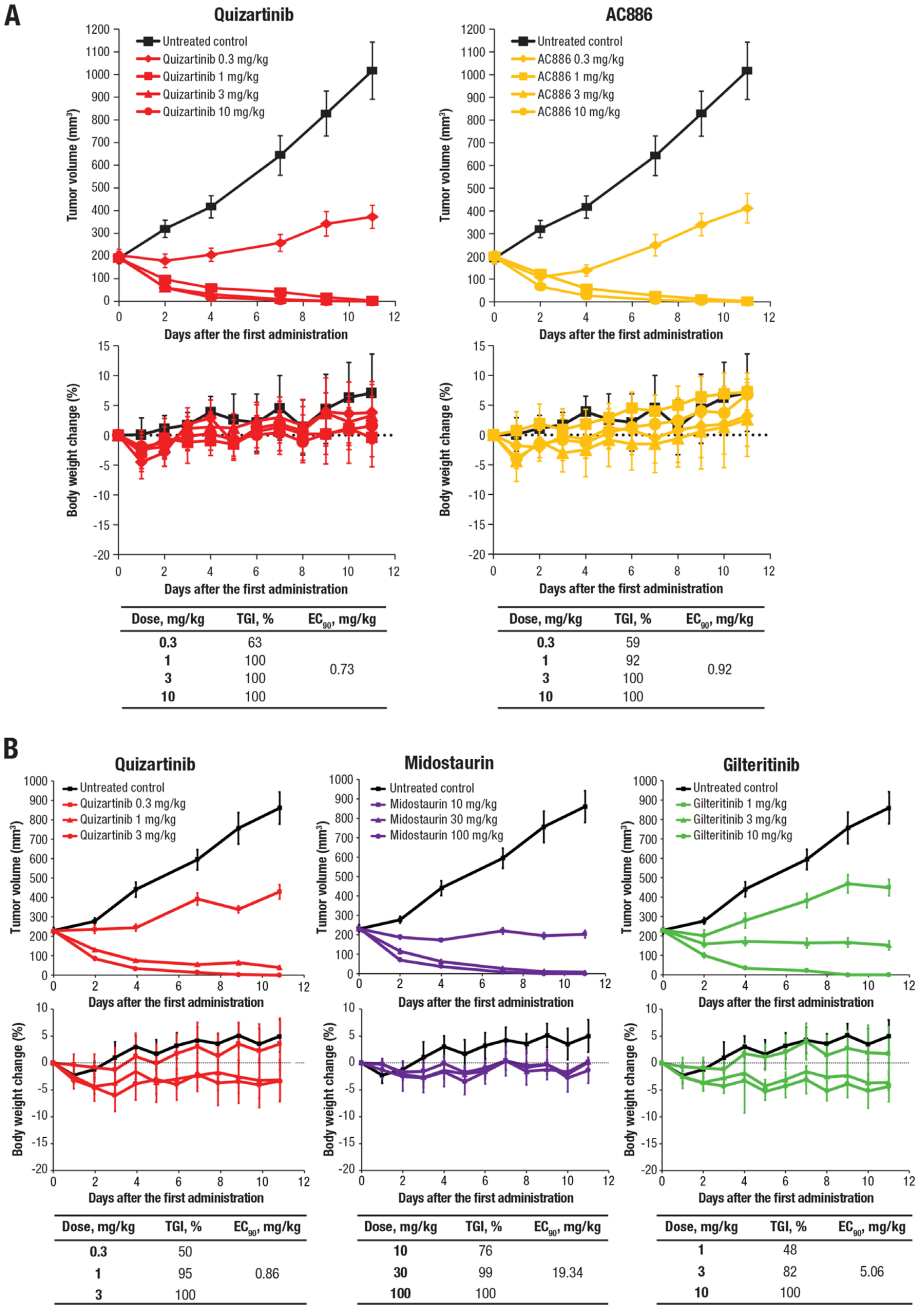
Quizartinib and AC886 exerted potent antitumor activity in the MV4-11 xenograft model. (**A**) Quizartinib and AC886 and (**B**) quizartinib, midostaurin, and gilteritinib were administered to mice bearing MV4-11 tumors. The upper graphs show tumor volume over time, with each data point and bar representing the mean and standard error of the estimated tumor volume in each group, respectively (*n* = 6). The lower graphs show change in body weight, with each data point and bar representing the mean and standard deviation of the percentage of body weight change in each group, respectively (*n* = 6).

The antileukemic effects of quizartinib and AC886 were assessed in a mouse xenograft model in which MV4-11 cells were injected into NOD/SCID mice. Oral administration of quizartinib or AC886 inhibited tumor growth at doses ranging from 0.3 to 10 mg/kg in a dose-dependent manner (each dose *P*<.0001 vs control), and both compounds had similar inhibitory effects, with 90% effective concentration (EC_90_) values of 0.73 and 0.92 mg/kg, respectively, without a significant impact on body weight (Figure 3A). AC886 has been identified as the major plasma metabolite of quizartinib and is rapidly converted from quizartinib in humans [27, 28]. To evaluate the pharmacokinetic profile of AC886 after quizartinib dosing in mice, quizartinib was orally administered at a dose of 1 mg/kg. In mice, the plasma concentration of quizartinib reached a maximum concentration 2 hours after administration. AC886 was detected 1 hour after quizartinib dosing, reaching its peak plasma concentration at 6 hours, suggesting that quizartinib is also rapidly converted to AC886 in mice (Supplementary Figure 2). Quizartinib demonstrated greater tumor growth inhibition than midostaurin and gilteritinib based on EC_90_ values (Figure 3B), using midostaurin and gilteritinib doses based on previous studies [29, 30].

### Quizartinib maintained inhibitory activity on the viability of midostaurin-resistant cells with *RAS* mutations

**Figure 4:**
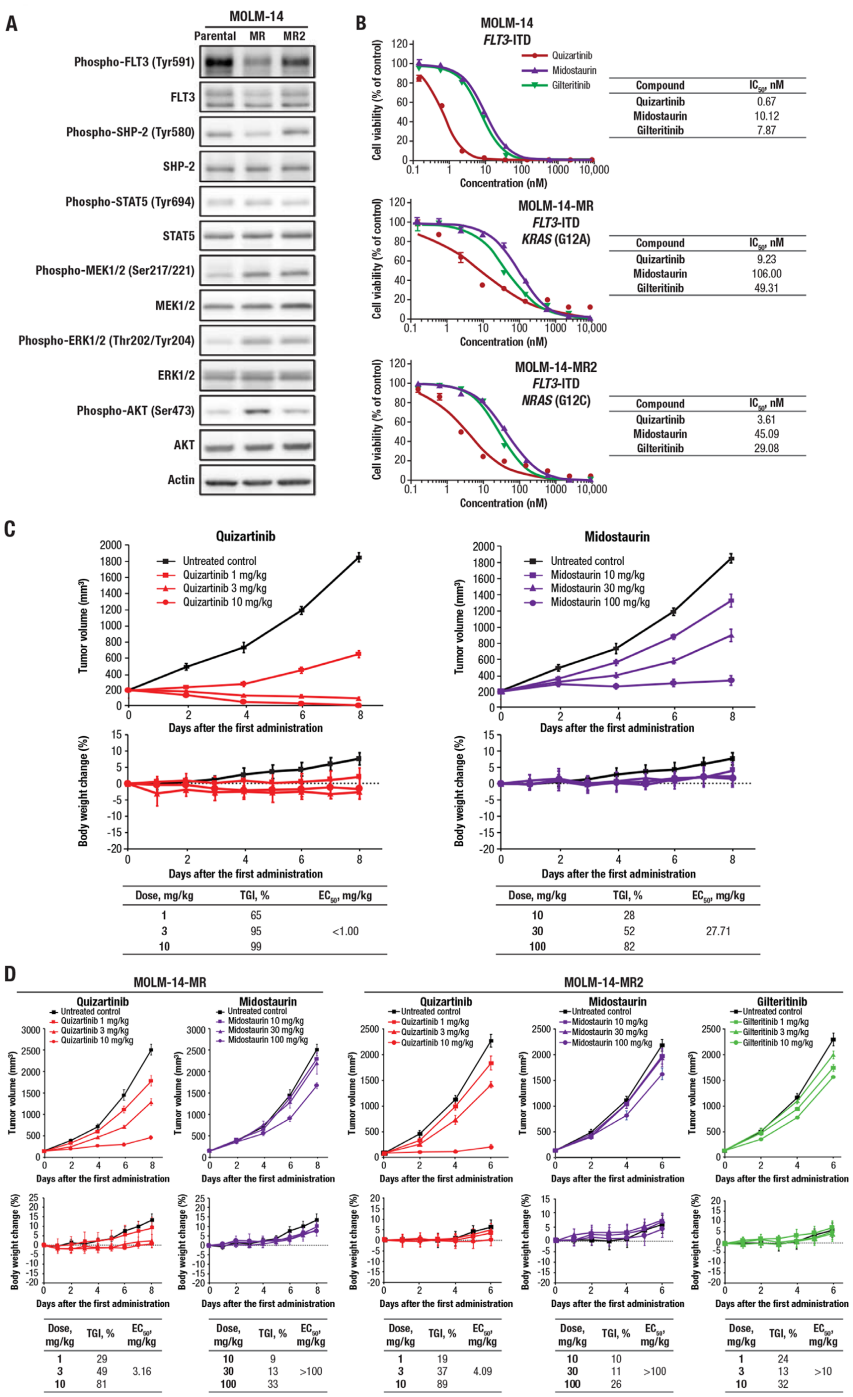
Quizartinib suppressed cell growth and had potent antitumor activity against midostaurin-resistant cells. (**A**) Cell lysates from MOLM-14, MOLM-14-MR, and MOLM-14-MR2 cells were obtained, and Western blot analysis was performed using antibodies against the indicated proteins. (**B**) Parental MOLM-14, MOLM-14-MR, and MOLM-14-MR2 cells were incubated with quizartinib, midostaurin, or gilteritinib for 3 days. Cell viability was assessed using the CellTiter-Glo 2.0 Assay (Promega Corporation, Madison, Wisconsin, USA) and plotted at each concentration. Each data point and bar represent the mean and standard deviation in each concentration group, respectively (*n* = 3). (**C**) Quizartinib or midostaurin was administered to mice bearing MOLM-14 tumors. (**D**) Quizartinib, midostaurin, and gilteritinib were administered to mice bearing MOLM-14-MR or MOLM-14-MR2 tumors. (C–D) The upper graphs show tumor volume over time, with each data point and bar representing the mean and standard error of the estimated tumor volume in each group, respectively (*n* = 6). The lower graphs show change in body weight, with each data point and bar representing the mean and standard deviation of the percentage of body weight change in each group, respectively (*n* = 6).

After long-term treatment of MOLM-14 cells with midostaurin, 2 mutations were identified that were associated with midostaurin resistance. Whole-genome sequencing revealed the mutations to be in *RAS*: *KRAS* (G12A; variant allele frequency [VAF], 37%) and *NRAS* (G12C; VAF, 55%) mutations, called MOLM-14-MR and MOLM-14-MR2 here, respectively. This is consistent with a previous finding of *RAS* mutations conferring resistance to the type I inhibitor lestaurtinib [31]. Compared with parental MOLM-14 cells, a mixed population of MOLM-14 cells with either wild-type *RAS* or midostaurin resistance–conveying *RAS* mutations showed increased phosphorylation of FLT3 downstream signaling molecules MEK, ERK, and AKT (Figure 4A). We next assessed the impact of midostaurin resistance on the ability of quizartinib, midostaurin, and gilteritinib to inhibit cell growth. In parental MOLM-14 cells, quizartinib inhibited cell viability with the IC_50_ value of 0.67 nM, while midostaurin and gilteritinib had IC_50_ values of 10.12 and 7.87 nM, respectively (Figure 4B). While the presence of *RAS* mutations resulted in 4- to 14-fold increases in IC_50_ values for all 3 compounds, quizartinib showed potent inhibition of cell growth against midostaurin-resistant cells with IC_50_ values of <10 nM (Figure 4B). We also investigated the impact of TKD mutations on FLT3 inhibitors. Quizartinib inhibited the cell viability of parental MOLM-13 cells, yet secondary FLT3 mutations D835Y and F691L conferred resistance to quizartinib (Supplementary Table 4). These results were consistent with previously reported findings and consistent with the mechanism of action of a type II inhibitor, and the same mutations were observed in patients with *FLT3*-ITD–positive AML with acquired resistance to quizartinib [32]. D835Y also conferred resistance to midostaurin, and F691L conferred resistance to both midostaurin and gilteritinib (Supplementary Table 4).

### Antileukemic activity of quizartinib in xenograft models of midostaurin-resistant cells

We assessed the impact of midostaurin resistance on the antileukemic activity of FLT3 inhibitors in the mouse xenograft model. Quizartinib demonstrated greater tumor growth inhibition than midostaurin against tumors derived from parental MOLM-14 cells (Figure 4C). When midostaurin-resistant MOLM-14-MR cells were used, quizartinib still showed tumor growth inhibition (50% effective concentration [EC_50_] of 3.16 vs <1.00 mg/kg with parental MOLM-14 tumors), while midostaurin had no significant tumor growth inhibition (EC_50_ of >100 vs 27.71 mg/kg with parental MOLM-14 tumors) (Figure 4D). Similar results were seen with midostaurin-resistant MOLM14-MR2 cells, with quizartinib and midostaurin having EC_50_ values of 4.09 and >100 mg/kg, respectively (Figure 4D). Additionally, gilteritinib had weak tumor inhibition using doses in the 1 to 10 mg/kg range in the xenograft model against MOLM-14-MR2–generated tumors.

## DISCUSSION

Here we provide preclinical data characterizing quizartinib, its active metabolite AC886, and other FLT3 inhibitors regarding kinase selectivity, extent of downstream effects of FLT3 inhibition, and activity in models of RAS-mediated midostaurin resistance. Quizartinib and AC886 were the most selective FLT3 inhibitors, binding fewer kinases with high affinity than the other FLT3 inhibitors evaluated, and demonstrated high affinity for the FLT3 kinase. Quizartinib and AC886 inhibited phosphorylation of FLT3 and downstream molecules, and transient exposure to quizartinib led to durable inhibition of FLT3. Additionally, quizartinib demonstrated the greatest activity in *in vitro* and *in vivo* models of RAS-mediated midostaurin resistance.

To our knowledge, this is the first report characterizing the kinase binding profile of the FLT3 inhibitors currently in clinical development, including second-generation inhibitors, under the same experimental conditions and using the KINOMEscan technology. In this study, quizartinib and its previously uncharacterized active metabolite AC886 inhibited the FLT3-ITD receptor with greater potency and selectivity than any of the other inhibitors. Kd values for nonmutant FLT3 binding obtained in this study were consistent with those previously reported for other FLT3 inhibitors [15, 16, 33]. In addition to having a similar kinase binding profile, quizartinib and AC886 both reduced the cell viability of *FLT3*-ITD cells to the same degree, and each independently had antitumor activity in a mouse xenograft model.

A notable observation of this study was that quizartinib retained antileukemic activity against midostaurin-resistant tumors. The IC_50_ values of quizartinib, midostaurin, and gilteritinib against established midostaurin-resistant MOLM-14 cells were 3.61 to 9.23, 45.09 to 106.00, and 29.08 to 49.31 nM, respectively. These IC_50_ values were measured in culture media. It has been reported that IC_50_ values of quizartinib, midostaurin, and gilteritinib in plasma, which are considered to be more clinically relevant, were approximately 20-, 160-, and 20-fold higher than those in culture media, respectively [34, 35]. Therefore, plasma IC_50_ values of quizartinib, midostaurin, and gilteritinib against midostaurin-resistant MOLM-14 cells could be estimated to be approximately 72 to 180, 7200 to 17,000, and 580 to 980 nM, respectively. Since mean trough concentrations after repeated quizartinib dosing for 15 days in humans were approximately 260 ng/mL (460 nM) for 40 mg once a day and 440 ng/mL (780 nM) for 60 mg once a day [36], quizartinib could maintain antileukemic activity against midostaurin-resistant tumors. However, mean trough concentrations of midostaurin and gilteritinib were reported to be approximately 900 to 3600 ng/mL (1600–3600 nM) for midostaurin 50 mg twice a day and 230 ng/mL (410 nM) for gilteritinib 120 mg once a day [29, 37], which seem to be lower than the estimated plasma IC_50_ values of these compounds against the midostaurin-resistant MOLM-14 cells.

**Figure 5:**
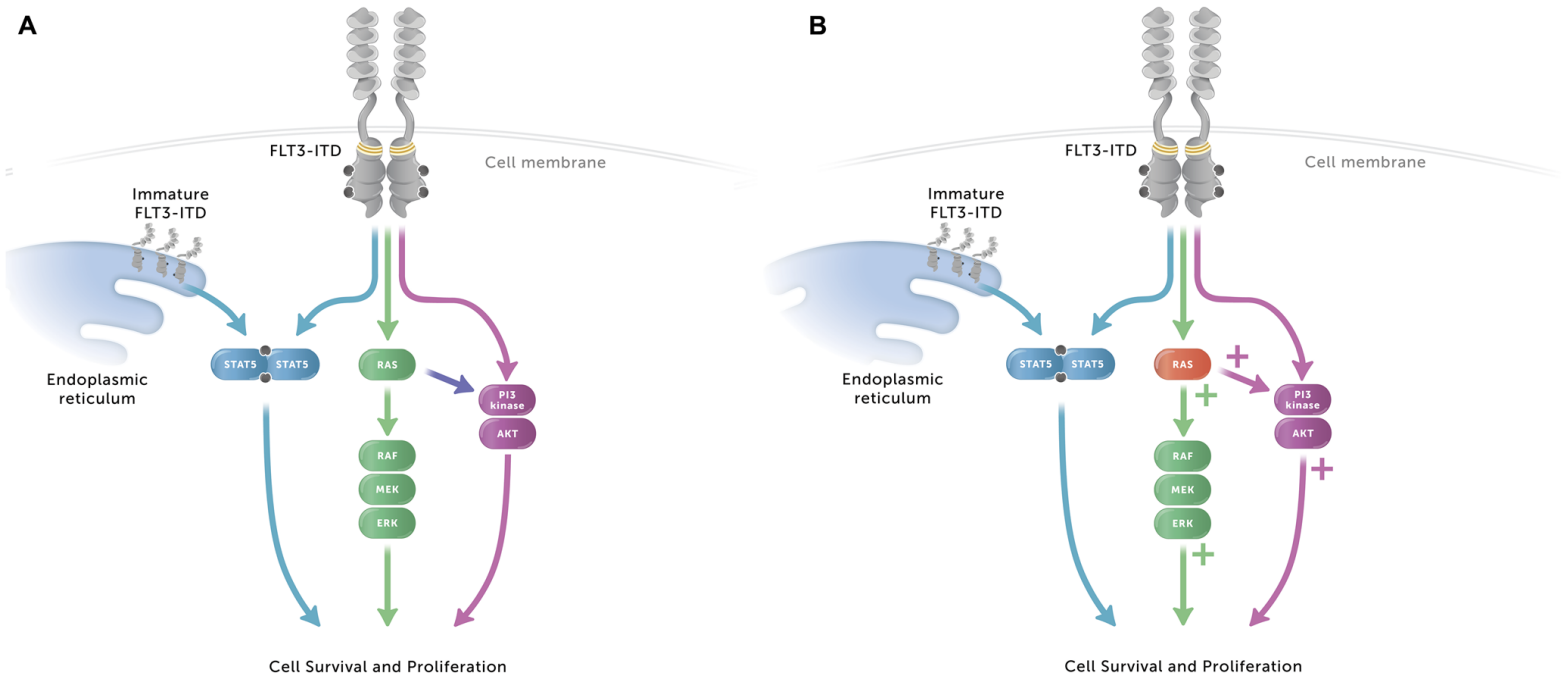
Signaling pathways in AML cells with *FLT3*-ITD with or without *RAS* mutation granting midostaurin resistance. (**A**) The *FLT3*-ITD mutation constitutively activates FLT3 signaling, independent of FLT3 ligand. FLT3 signals through the PI3 kinase/AKT pathway, the MEK/ERK pathway, and through STAT5, both from the membrane and as an immature form from the endoplasmic reticulum. (**B**) When cells with *RAS* mutations associated with midostaurin resistance make up part of the cell population, upregulation of the MEK/ERK and PI3 kinase/AKT pathways occurs, along with increased cell survival and proliferation.

There are multiple mechanisms by which resistance to midostaurin can develop, including secondary mutations in *FLT3* that alter the binding affinity of midostaurin to FLT3 and activation of downstream signaling molecules, leading to conservation of FLT3 signaling in the presence of the inhibitor [31, 38, 39]. As *FLT3*-ITD mutations constitutively activate FLT3 signaling, acting through the PI3 kinase/AKT pathway, the MEK/ERK pathway, or STAT5 (Figure 5A), activation of downstream molecules in these pathways are potential mechanisms for maintaining signaling during FLT3 inhibitor exposure. In this study, *NRAS* and *KRAS* mutations in MOLM-14 cells were associated with increased phosphorylation of FLT3 downstream molecules MEK1/2, ERK1/2, and AKT and increased midostaurin resistance (Figure 5B). Activation of the signaling pathway through mutation of *NRAS* has previously been described as a mechanism of midostaurin resistance [31]. One possible explanation of this finding is that less potent type I FLT3 inhibitors allow for low-level FLT3 signaling that can be amplified and overcome by *RAS* mutations, but these downstream mutations have a lesser impact on highly potent type II inhibitors that more completely inhibit signaling. The VAFs of *RAS* for MOLM-14-MR and MOLM-14-MR2 were 37% and 55%, respectively, and it would be of interest to assess the impact of *RAS* mutations with higher VAFs on FLT3 inhibitor resistance. In addition to potent inhibition of FLT3 phosphorylation, quizartinib had sustained inhibition of FLT3 signaling, observed up to 24 hours following drug removal here and as previously described [26]. Interestingly, this effect was not observed with gilteritinib. We are currently developing *FLT3*-ITD cell lines with resistance to gilteritinib in order to carry out similar studies regarding cross-resistance with quizartinib and other FLT3 inhibitors. Gilteritinib has been reported to maintain activity against secondary mutations in the TKD (D835Y) [30, 34], yet *RAS* mutations were a common mechanism of acquired resistance to gilteritinib in patients with *FLT3*-mutated AML [40].

Quizartinib retained both *in vitro* activity against the proliferation of mixed *FLT3*-ITD cell lines with parental or midostaurin resistance–conveying *NRAS* and *KRAS* mutations and *in vivo* activity against midostaurin-resistant *FLT3*-ITD tumors with EC_50_ values of approximately 3 to 4 mg/kg compared with >100 mg/kg for midostaurin and >10 mg/kg for gilteritinib (*NRAS* mutated only). These results suggest the potential value of quizartinib for the treatment of patients with *FLT3*-ITD mutations for whom a previous midostaurin treatment regimen failed, but further clinical investigation is needed.

In conclusion, we have demonstrated the high affinity and selectivity that quizartinib and its active metabolite AC886 have for FLT3 and that quizartinib maintains preclinical antitumor activity against midostaurin-resistant tumor models. Additional clinical trials will be needed to clarify and optimize the role of quizartinib in the treatment of patients with relapsed or refractory AML who have previously been treated with midostaurin.

## MATERIALS AND METHODS

### Reagents and cell culture

Quizartinib dihydrochloride and its active metabolite AC886 were prepared by Daiichi Sankyo, Inc. Midostaurin, gilteritinib, crenolanib, and sorafenib were synthesized by Daiichi Sankyo Co, Ltd, for research purposes only. Each compound was dissolved in dimethyl sulfoxide (DMSO), and DMSO was used as the control treatment for *in vitro* experiments.

Human AML cell lines MV4-11, Kasumi-1, HL-60, and THP-1 were purchased from the American Type Culture Collection (Manassas, Virginia, USA). MV4-11 cells were cultured in Iscove’s Modified Dulbecco’s Medium (IMDM; Thermo Fisher Scientific, Waltham, Massachusetts, USA) supplemented with 10% (vol/vol) heat-inactivated fetal bovine serum (FBS; GE Healthcare Ltd, Chicago, Illinois, USA). Kasumi-1 cells were cultured in RPMI-1640 medium (FUJIFILM Wako Pure Chemical Corporation, Osaka, Japan) supplemented with 20% (vol/vol) heat-inactivated FBS. HL-60 cells were cultured in IMDM supplemented with 20% (vol/vol) heat-inactivated FBS. THP-1 cells were cultured in RPMI-1640 medium supplemented with 20% (vol/vol) heat-inactivated FBS and 0.05 mM StemSure 2-Mercaptoethanol Solution (FUJIFILM Wako Pure Chemical Corporation). Human AML cell lines, MOLM-13, MOLM-14, EOL-1, and OCI-AML3, were purchased from Leibniz-Institut DSMZ-Deutsche Sammlung von Mikroorganismen und Zellkulturen GmbH. MOLM-13, MOLM-14, and EOL-1 cells were cultured in RPMI-1640 medium supplemented with 10% (vol/vol) heat-inactivated FBS, and OCI-AML3 cells were cultured in Minimum Essential Medium α (Thermo Fisher Scientific) supplemented with 20% (vol/vol) heat-inactivated FBS.

Following long-term treatment of MOLM-14 cells with midostaurin, 2 midostaurin-resistant MOLM-14 lines (MOLM-14-MR and MOLM-14-MR2) emerged. Whole-exome sequencing revealed that *KRAS* (G12A; VAF, 37%) and *NRAS* (G12C; VAF, 55%) mutations occurred in MOLM-14-MR and MOLM-14-MR2, respectively. After long-term treatment of MOLM-13 cells with quizartinib, 2 quizartinib-resistant MOLM-13 lines (MOLM-13-QR and MOLM-13-QR2) were discovered. MOLM-13-QR contained *FLT3* (D835Y; VAF, 32%) and MOLM-13-QR2 contained *FLT3* (F691L; VAF, 29%) mutations.

### Kinase binding affinity assays

KINOMEscan kinase binding assays were performed at a Eurofins laboratory as previously described [14, 41].

### Cell viability assays

Aliquots of cells were treated with each inhibitor at concentrations ranging from 0.153 to 10,000 nM and cultured in the medium for 3 days. The amount of ATP in viable cells was quantified as a luminescent signal using the CellTiter-Glo 2.0 Assay (Promega Corporation, Madison, Wisconsin, USA) and EnVision (PerkinElmer Inc, Waltham, Massachusetts, USA) to determine the number of viable cells according to the manufacturers’ instructions.

### FLT3 signaling pathway analysis

MV4-11, MOLM-14, MOLM-14-MR, and MOLM-14-MR2 cells were used for FLT3 signaling pathway analysis. MV4-11 cells were treated with quizartinib or AC886 at concentrations ranging from 0.0625 to 8 nM for 2 hours. After centrifugation, the cell pellets were washed by ice-cold Dulbecco’s Phosphate-Buffered Saline (FUJIFILM Wako Pure Chemical Corporation), suspended, and lysed in cell lysis buffer (Cell Signaling Technology, Inc, Danvers, Massachusetts, USA) containing cOmplete (Roche Diagnostics, Basel, Switzerland) and PhosSTOP (Roche Diagnostics). After centrifugation, supernatants were used as cell lysates. The cell lysates were resolved by sodium dodecyl sulfate–polyacrylamide gel electrophoresis, followed by immunoblotting. Phospho-FLT3 (Tyr591), total FLT3, phospho-SHP-2 (Tyr580), total SHP-2, phospho-STAT5 (Tyr694), phospho-MEK1/2 (Ser217/221), total MEK1/2, phospho-ERK1/2 (Thr202/Tyr204), total ERK1/2, phospho-AKT (Ser473), total AKT, and β-actin were probed with corresponding rabbit antibodies (Catalog No. 3461, 3462, 3703, 3397, 4322, 9121, 9126, 4370, 4695, 4058, 9272, and 4970, respectively; Cell Signaling Technology, Inc) as primary antibodies. Total STAT5 was also probed with corresponding rabbit antibody (Catalog No. ab194898; Abcam plc, Cambridge, UK). Horseradish peroxidase–conjugated goat antirabbit IgG (Cell Signaling Technology, Inc) was used as a secondary antibody. Immunoblotting detection was performed with Luminata Forte Western HRP Substrate (Merck KGaA, Darmstadt, Germany) and Fusion FX7 (Vilber Lourmat, Collegien, France) or ImageQuant LAS 4000 mini (GE Healthcare) according to the manufacturers' instructions.

### FLT3 inhibitor washout experiment

MOLM-14 cells in RPMI-1640/10% FBS were adjusted to a concentration of 1 million cells/mL. Quizartinib, AC886, gilteritinib, midostaurin, crenolanib, and sorafenib in DMSO were diluted in culture medium. An aliquot of cells was removed (baseline). Cells were incubated for 1 or 2 hours in a CO_2_ incubator at 37°C with quizartinib, AC886, gilteritinib, midostaurin, crenolanib, or sorafenib, all at a concentration of 20 nM, or DMSO control. Another aliquot of cells was removed (0-hour control). Cells were then pelleted, washed once in conditioned medium, and resuspended in conditioned medium at 1 million cells/mL. Aliquots of cells were removed after 1, 2, 3, 4, or 24 hours of incubation.

Upon removal from culture, cells were pelleted, washed in phosphate-buffered saline, lysed in detergent buffer, and subject to immunoprecipitation and Western blotting. FLT3 was immunoprecipitated with rabbit anti-FLT3 (S-18; Santa Cruz, Dallas, Texas, USA), followed by Western blotting with antiphosphotyrosine (4G10; Millipore, Burlington, Massachusetts, USA). Whole-cell lysate was directly analyzed by Western blotting for ERK and phospho-ERK (Cell Signaling Technology, Inc). Alternatively, the cell lysates were analyzed as described in “FLT3 signaling pathway analysis.”

### *In vivo* xenograft mouse models

Specific pathogen–free female NOD SCID mice (NOD/ShiJic-scid Jcl), aged 5 weeks, for the MV4-11 xenograft model, and specific pathogen–free female SCID mice (Fox Chase SCID C.B-17/Icr-scid/scidJcl), aged 6 weeks, for the MOLM-14 xenograft model, were purchased from CLEA Japan, Inc (Tokyo, Japan).

The *in vitro* cultured MV4-11 cells were transplanted subcutaneously into female NOD SCID mice at 1 × 10^7^ cells/mouse, and MOLM-14, MOLM-14-MR, or MOLM-14-MR2 cells were transplanted into female SCID mice at 5 × 10^6^ cells/mouse. When the average estimated tumor volume was >100 mm^3^, mice were randomly grouped on the basis of the estimated tumor volume by using the management system for animal experimental data (SMAD; JMACSOFT Corp, Nagaoka, Japan). The grouping day was defined as day 0, and each group consisted of 6 mice that received each treatment. Quizartinib and AC886 were dissolved in 22% hydroxypropyl-β-cyclodextrin (Tokyo Chemical Industry Co, Ltd, Tokyo, Japan). Midostaurin and gilteritinib were dissolved in 0.5% (wt/vol) sterilized methyl cellulose 400 solution (FUJIFILM Wako Pure Chemical Industries, Ltd). Each compound was orally administered once per day at indicated doses as a free base. The tumor length (L) and width (W) in millimeters were measured using a digital caliper (CD-15CX; Mitutoyo Corporation, Kanagawa, Japan), and the mouse body weight was measured with a digital balance for animals (UW2200H; Shimadzu Corporation, Kyoto, Japan). The estimated tumor volume of each mouse was automatically calculated in the SMAD according to the following equation: Estimated tumor volume (mm^3^) = 1/2 × L × W^2^. Mean estimated tumor volume was determined for the compound-treated (T) and untreated control (C) groups. Tumor growth inhibition was automatically calculated in the SMAD according to the following equation: Tumor growth inhibition (%) = (1 − T/C) × 100. All animal experimental procedures were performed according to the guidelines of the Institutional Animal Care and Use Committee of Daiichi Sankyo Co, Ltd.

### Statistical analysis

For estimated change in tumor volume with each inhibitor, a parametric Dunnett test was conducted between the T and C groups to evaluate the antitumor activity of the compound. A 2-sided *P* value of <0.05 was considered statistically significant. Hypothesis testing of Spearman rank correlation coefficient (the null hypothesis correlation coefficient is 0) was conducted among the T and C groups to evaluate the dose-dependent antitumor activity of the compound. All statistical analyses were performed using REDPOST/BI (SAS System Release 9.2; SAS Institute Inc, Cary, North Carolina, USA).

The IC_50_ (nM), the EC_50_ (mg/kg), and the EC_90_ (mg/kg) were estimated using REDPOST/BI.

## SUPPLEMENTARY MATERIALS





## Abbreviations

AML: acute myeloid leukemia
DMSO: dimethyl sulfoxide
EC_50_: 50% effective concentration
EC_90_: 90% effective concentration
FBS: fetal bovine serum
FLT3: FMS like tyrosine kinase 3
IC_50_: 50% inhibitory concentration
IMDM: Iscove’s Modified Dulbecco’s Medium
ITD: internal tandem duplication
TKD: tyrosine kinase domain
VAF: variant allele frequency
