# Supplementary material for: Quizartinib, a selective FLT3 inhibitor, maintains antileukemic activity in preclinical models of RAS-mediated midostaurin-resistant acute myeloid leukemia cells
Source: Oncotarget. 2020 Mar 17;11(11):943–55. doi: 10.18632/oncotarget.27489 (PMC7082118; doi:10.18632/oncotarget.27489)
Supplement: Supplementary file 1 [file oncotarget-11-943-s001.pdf]

Quizartinib, a selective FLT3 inhibitor, maintains antileukemic activity in preclinical models of RAS-mediated midostaurin-resistant acute myeloid leukemia cells

SUPPLEMENTARY MATERIALS

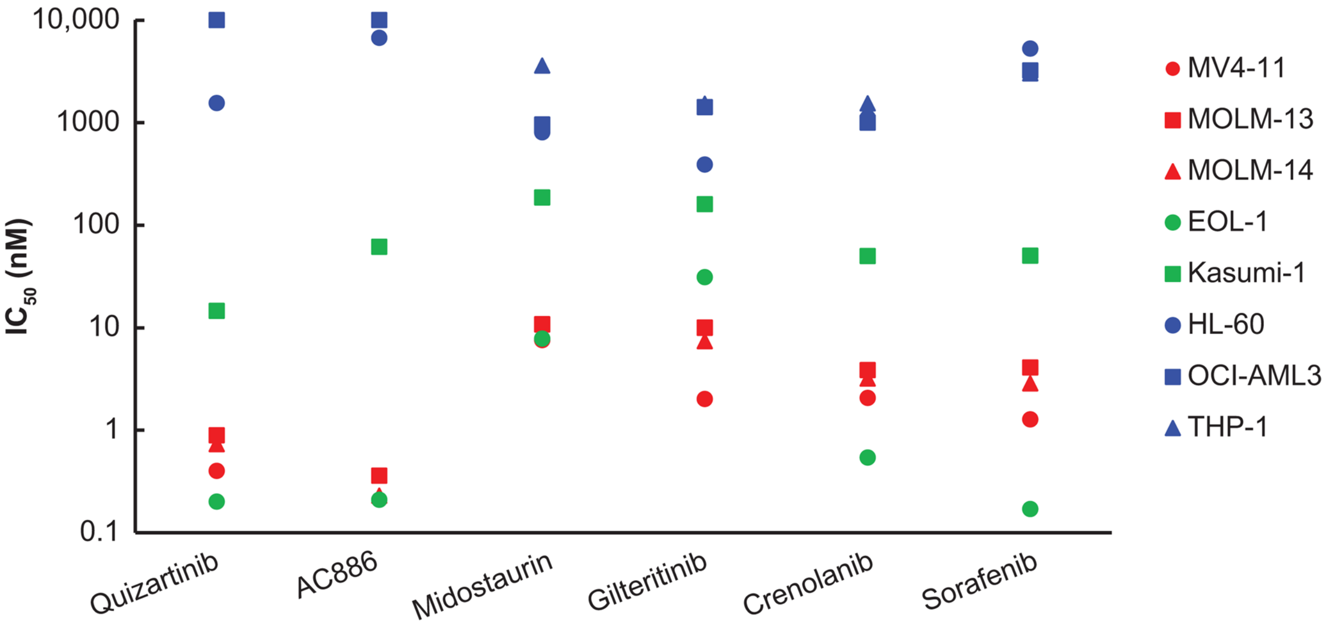

Supplementary Figure 1: The effect of quizartinib and other FLT3 inhibitors on the viability of AML cells. Cells were treated with each inhibitor and cultured in the medium for 3 days. The amount of ATP in viable cells was quantified as a luminescent signal using the CellTiter-Glo 2.0 Assay (Promega Corporation, Madison, Wisconsin, USA) and EnVision (PerkinElmer Inc, Waltham, Massachusetts, USA) to determine the number of viable cells according to the manufacturer's instructions. Red symbols represent AML cells with *FLT3*-ITD mutation, green symbols represent wild-type *FLT3* AML cells with mutations in the *PDGFRα* or *KIT* genes, and blue symbols represent wild-type *FLT3* AML cells. All IC_{50} values ≥10,000 nM were plotted as 10,000 nM.

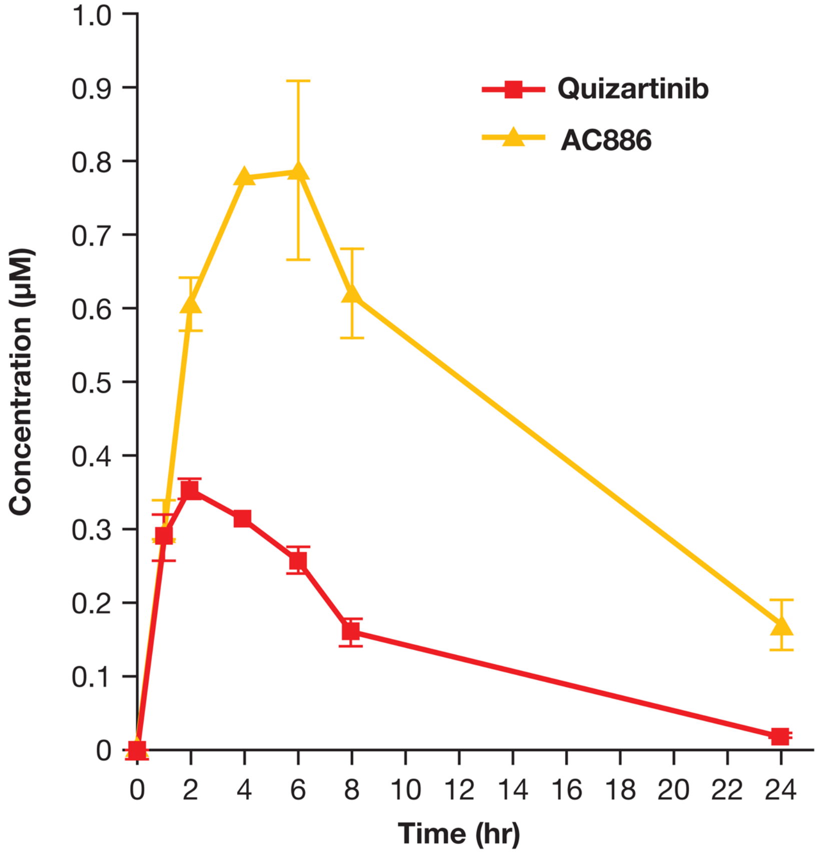

Supplementary Figure 2: Pharmacokinetics profile of quizartinib and its metabolite, AC886, after oral administration of quizartinib to mice. Quizartinib was administered to MV4-11 xenograft model mice at a dose of 1 mg/kg. Blood samples were collected at 1, 2, 4, 6, 8, and 24 hours after dosing and centrifuged to separate the supernatant as plasma. Plasma concentrations of quizartinib and AC886 were quantified by liquid chromatography–tandem mass spectrometry. Results are indicated as mean ± standard deviation of the concentration of each group (n = 3 at each time point except for n = 2 at 4 hours).

Supplementary Table 1: Binding affinity of quizartinib, AC886, and other FLT3 inhibitors to select kinases*

Kinase	Kd, nM					
	Quizartinib	AC886	Midostaurin	Gilteritinib	Crenolanib	Sorafenib
FLT3	3.3	1.1	7.9	1.0	0.28	5.9
KIT	3.9	0.93	330	86	36	26
CSF1R/FMS	32	19	360	46	7.2	38
PDGFRα	33	11	260	53	0.67	12
PDGFRβ	11	3.7	33	240	2.9	21
FLT1	110	190	1200	380	1500	35
FLT4	38	51	150	150	180	25
KDR/VEGFR2	98	60	1200	100	320	23
RET	11	19	82	11	120	4.4

*For binding affinities of FLT3 inhibitors to all 404 nonmutant kinases tested, see Supplementary Appendix.

Supplementary Table 2: Off-target inhibitory activity of quizartinib and AC886

Target molecule	Quizartinib<br>% inhibition at 10 μM	AC886<br>% inhibition at 10 μM
Cholinesterase, acetyl, ACES	30	64
Sodium channel, site 2	92	72
Tachykinin NK1	80	25
Transporter, adenosine	36	53

SafetyScreen enzymatic and binding assays on off-target molecules such as G-protein–coupled receptors, transporters, ion channels, nuclear receptors, and enzymes were performed at a Eurofins laboratory. The assays were performed in duplicate at a concentration of 10 μM for each compound on 87 target molecules. Significant responses, defined as ≥50% inhibition, are shown here.

Supplementary Table 3: IC_{50} values of quizartinib, AC886, and other FLT3 inhibitors in AML cells

Cell line	FLT3 status and other genetic characteristics	IC_{50}, nM					
		Quizartinib	AC886	Midostaurin	Gilteritinib	Crenolanib	Sorafenib
MV4-11	*FLT3*-ITD	0.40	0.21	7.56	2.01	2.06	1.27
MOLM-13	*FLT3*-ITD	0.89	0.36	10.72	10.00	3.86	4.10
MOLM-14	*FLT3*-ITD	0.73	0.23	8.73	7.41	3.19	2.85
EOL-1	Wild-type *FLT3* with FIP1L-PDGFRα	0.20	0.21	7.86	30.91	0.54	0.17
Kasumi-1	Wild-type *FLT3* with KIT (N822K)	14.45	61.49	185.16	159.96	49.67	50.39
HL-60	Wild-type *FLT3*	1549.58	6742.95	806.24	390.83	1107.47	5285.15
OCI-AML3	Wild-type *FLT3*	>10000	>10000	955.33	1417.04	1001.70	3231.88
THP-1	Wild-type *FLT3*	>10000	>10000	3604.98	1527.71	1533.64	3029.57

Supplementary Table 4: Impact of FLT3 inhibitors on parental MOLM-13 cells and MOLM-13 cell lines with acquired quizartinib resistance

Cell line	FLT3 status and acquired gene alteration	Quizartinib IC_{50}, nM	Midostaurin IC_{50}, nM	Gilteritinib IC_{50}, nM
MOLM-13	*FLT3*-ITD	0.62	7.40	7.89
MOLM-13-QR	*FLT3*-ITD<br>FLT3 (D835Y)	43.51	38.57	9.68
MOLM-13-QR2	*FLT3*-ITD<br>FLT3 (F691L)	52.71	24.20	43.02

Supplementary Appendix: Binding affinity of quizartinib, AC886, and other FLT3 inhibitors to all 404 nonmutant kinases. See Supplementary Appendix
